# Consensus cluster analysis of apoptosis-related genes in patients with osteoarthritis and their correlation with immune cell infiltration

**DOI:** 10.3389/fimmu.2023.1202758

**Published:** 2023-10-04

**Authors:** Enming Yu, Mingshu Zhang, Gongping Xu, Xiaoqi Liu, Jinglong Yan

**Affiliations:** Department of Orthopedics, The Second Affiliated Hospital of Harbin Medical University, Harbin, China

**Keywords:** osteoarthritis, apoptosis-related genes, differentially expressed genes, immune infiltration, bioinformatics analysis

## Abstract

**Background:**

Osteoarthritis (OA) progression involves multiple factors, including cartilage erosion as the basic pathological mechanism of degeneration, and is closely related to chondrocyte apoptosis. To analyze the correlation between apoptosis and OA development, we selected apoptosis genes from the differentially expressed genes (DEGs) between OA and normal samples from the Gene Expression Omnibus (GEO) database, used lasso regression analysis to identify characteristic genes, and performed consensus cluster analysis to further explore the pathogenesis of this disease.

**Methods:**

The Gene expression profile datasets of OA samples, GSE12021 and GSE55235, were downloaded from GEO. The datasets were combined and analyzed for DEGs. Apoptosis-related genes (ARGs) were collected from the GeneCards database and intersected with DEGs for apoptosis-related DEGs (ARDEGs). Least absolute shrinkage and selection operator (LASSO) regression analysis was performed to obtain characteristic genes, and a nomogram was constructed based on these genes. A consensus cluster analysis was performed to divide the patients into clusters. The immune characteristics, functional enrichment, and immune infiltration statuses of the clusters were compared. In addition, a protein–protein interaction network of mRNA drugs, mRNA-transcription factors (TFs), and mRNA-miRNAs was constructed.

**Results:**

A total of 95 DEGs were identified, of which 47 were upregulated and 48 were downregulated, and 31 hub genes were selected as ARDEGs. LASSO regression analysis revealed nine characteristic genes: growth differentiation factor 15 (*GDF15)*, *NAMPT*, *TLR7*, *CXCL2*, *KLF2*, *REV3L*, *KLF9*, *THBD*, and *MTHFD2*. Clusters A and B were identified, and *neutrophil activation* and *neutrophil activation involved in the immune response* were highly enriched in Cluster B, whereas *protein repair* and p*urine salvage* signal pathways were enriched in Cluster A. The number of activated natural killer cells in Cluster B was significantly higher than that in Cluster A. *GDF15* and *KLF9* interacted with 193 and 32 TFs, respectively, and *CXCL2* and *REV3L* interacted with 48 and 82 miRNAs, respectively.

**Conclusion:**

ARGs could predict the occurrence of OA and may be related to different degrees of OA progression.

## 1 Introduction

Osteoarthritis (OA) mainly affects the articular cartilage and is the most common joint disease. It is characterized by joint pain, stiffness, hypertrophy, and limited activity. It is most common in weight-bearing joints, such as the knee, hip, cervical, and lumbar spine, as well as the proximal and distal finger joints (1). It is the most prevalent among middle-aged and older individuals. Among people aged >65 years, the incidence of the disease is as high as 50% (2). The following clinical manifestations support the diagnosis of OA: Age of onset ≥ 45 years; Persistent usage-related joint pain in one or a few joints; Early morning and Short-lived (≤ 30 minutes) stiffness (3). According to X-ray findings, OA can be classified into 5 grades as follows: 0-None; 1-Doubtful; 2-Minimal; 3-Moderate; 4-Severe (4). The differential diagnosis for OA mainly depends on the location of the affected site and the presence other systemic symptoms. The differential diagnosis includes rheumatoid arthritis, psoriatic arthritis, crystalline arthritis, hemochromatosis, infectious arthritis, and other soft-tissue abnormalities. This disease is a progressive disease that eventually causes degeneration, fibrosis, rupture, defects, and damage to the entire articular surface of the joint, considerably affecting the lives of patients. If not treated promptly, there may be a risk of disability for patients (5). Owing to the fact that the etiology and pathogenesis of OA are not yet fully understood, symptomatic treatment can only be used to delay the progression of the disease, alleviate the symptoms of patients, correct deformities through surgery in the late stage, and improve the function of affected limbs (6). Therefore, OA pathogenesis requires further investigation.

Currently, most researchers believe that OA is the result of a combination of mechanical and biological factors leading to an imbalance in the degradation and anabolism of articular chondrocytes, extracellular matrix (ECM), and subchondral bone (7). Apoptosis is a process of programmed cell death controlled by genes. The human body maintains the homeostasis of various system tissues by clearing the dead cells and metabolites. However, if the apoptosis process is disrupted, it is directly or indirectly related to the occurrence of many diseases, such as tumors, autoimmune diseases, local injuries, etc. (8). Apoptosis in the cartilage tissue related to matrix degradation and calcification has been detected in OA cartilage, indicating that apoptosis plays a role in OA pathogenesis (9).

To comprehensively understand the role of apoptosis and immune mechanisms in the occurrence and development of OA, we analyzed transcriptome data from the GEO database, selected characteristic apoptosis-related genes (ARGs) that could predict OA occurrence, and attempted to cluster patients with OA based on these genes. We analyzed the immune characteristics, biological pathways, and immune infiltration between different clusters and searched for drugs and molecules that inhibit OA occurrence.

## 2 Materials and methods

### 2.1 Data downloading and initial preparation

OA-related chip sequencing data and corresponding clinical sample information were downloaded from the GSE12021 (10) and GSE55235 (11) datasets in the GEO database. The sample source was *Homo sapiens* and the two dataset sequencing platforms were GPL96 and GPL97. The data set GSE12021 contains 33 samples in total, including 13 normal samples and 20 samples from patients with OA; the data set GSE55235 contains 20 samples in total, including 10 normal samples and 10 samples from patients with OA. We integrated the above sets of data expression into one data set, which was named as the combined data or combined gene expression. Then, the R package “sva” (12) was used to correct the batch effect between different data sets and log2 (X+1) standardization was performed. Standardized data were used as the basis for subsequent analyses.

### 2.2 Apoptosis-related differentially expressed genes

A total of 3,576 ARGs were collected from the GeneCards database (13) and other documents. The differential gene analysis between OA samples and normal samples was conducted using the R package “limma” (14). Significant DEGs were screened using the absolute value of the log2Fold change (log2FC) > 1 and *P*< 0.05. The upregulated genes were defined as log2FC > 1 and *P*< 0.05, and the downregulated DEGs were defined as log2FC< - 1 and *P<* 0.05. The DEG expression results are displayed on a volcano map and a heatmap. The ARGs and DEGs are intersected as hub genes called ARDEGs.

### 2.3 Least absolute shrinkage and selection operator regression analysis and risk model construction

To accurately screen for biomarkers related to OA, we conducted dimensionality reduction screening using the LASSO model with 1,000 iterations (15). The objective function of the LASSO regression model was as follows:


$$min\int^{\;}(\alpha_{0},\alpha |X_{i},Y_{i}+\;\lambda {\left|\left|\alpha \right|\right|}_{1})$$

where λ represents the penalty coefficient, which can be selected through 10-fold cross-validation for the chosen λ; ||α||_1_ is defined as the sum of the absolute values of each vector element. LASSO regression was implemented through the R package “glmnet” (16). A risk-scoring formula was established by weighing each normalized gene expression value with the penalty coefficient of the characteristic gene.


$$\text{riskScore }=\;\sum_{\text{i}}\text{Coefficient }\left({\text{gene}}_{\text{i}}\right)*\text{mRNA Expression }\left({\text{ gene}}_{\text{i}}\right)$$

Subsequently, a nomogram model was constructed based on selected candidate ARDEGs to predict OA.

### 2.4 Consensus clustering method and identification of apoptotic subtypes

Based on the expression data of the characteristic genes in the Combine data chipset with all the samples, apoptotic clusters were identified using the R package “ConsensusClusterPlus” (17). The R package “limma” was used again to analyze the differential genes of different apoptotic subtypes in the combined data set, with |log2FC| > 1 and *P<* 0.05 as the differential gene-screening criteria. DEGs with |log2FC| > 4 and *P*< 0.05 were considered upregulated, whereas those with |log2FC|< -3 and *P*< 0.05 were considered downregulated. The results are displayed using a volcano map.

### 2.5 Gene function enrichment analysis by GO and single-sample gene set enrichment analysis

GO enrichment analysis (18) is a common method for conducting large-scale functional enrichment studies on biological processes (BPs), molecular functions (MFs), and cellular components (CCs). It was conducted for all the ARDEGs through the “clusterProfiler” package (19). To analyze the differences in BPs between subgroups based on gene expression data, we used ssGSEA (20), which is a computational method that analyzes whether a specific gene set shows statistical differences between two biological states. It is commonly used to estimate changes in pathways and BPs in expression dataset samples, with a P value< 0.05 considered to be statistically significant.

### 2.6 Correlation analysis and chromosome location between genes

To explore the correlation among ARDEGs, spearman analysis was performed through the R package “cowplot” (21), and heat maps, scatter maps, and correlation curves were drawn. P values< 0.05 imply that the genes have a strong correlation. The R package “RCircos” (22) was used to draw the location map of the key genes in the chromosome with the location information of genes downloaded from the ENSEMBL database (23).

### 2.7 CIBERSORT for the infiltration state of immune cells

CIBERSORT (https://cibersort.stanford.edu/) (24) is an R/webpage version tool that deconvolutes the expression matrix of human immune cell subtypes based on the principle of linear support vector regression. The expression of 22 types of known immune cells was calculated and evaluated for infiltration status using the CIBERSORT algorithm, and the percentage difference of immune cells between the OA samples and the normal samples was tested using the Wilcoxon test, with a *P* value< 0.05 considered to be statistically significant.

### 2.8 Drug–gene interaction analysis

We searched the DGIdb database (Version 3.0.2, https://www.dgidb.org) (25) with ARDEG names as the input to predict any potential drugs or molecular compounds interacting with them. The results were visualized by a drug–gene interaction network using the Cytoscape software.

### 2.9 Construction of mRNA–miRNA and mRNA–transcription factor networks

MiRNAs (26) are a class of non-coding single-stranded RNA molecules encoded by endogenous genes, with a length of approximately 19–25 nucleotides, that play an essential regulatory role in the evolution of biological development. MiRNAs regulate the expression of target genes by participating in the post-transcriptional regulation of genes and play a crucial regulatory role in tumor occurrence and development, biological development, organ formation, epigenetic regulation, and viral defense. Typically, miRNAs have very complex regulatory networks and often one miRNA can regulate multiple target genes, whereas the same target gene can also regulate many miRNAs. To analyze the relationship between hub genes and miRNAs at the post-transcriptional stage, we obtained hub gene miRNAs from the Network Analyst database (27) and constructed an mRNA–miRNA regulatory network. TFs control gene expression by interacting with target genes during the post-transcriptional stage. To analyze the regulatory effect of TFs on hub genes, the targeted relationship between transcription factors and hub genes was retrieved from the Network Analyst database, and an interaction network between hub genes and TFs was constructed.

### 2.10 Statistical methods

All data were processed and analyzed using the R software (version 4.1.1). To compare two continuous variables that were distributed normally, an independent Student’s t-test was conducted. The Mann–Whitney U test was performed to determine the difference between non-normally distributed variables. Pearson’s correlation analysis was used to calculate the correlation coefficient (r) between the different genes, the Pearson’s correlation coefficient between two variables is defined as the quotient of the covariance and standard deviation between two variables:


$$\rho X,Y=\;\frac{cov\left(X,Y\right)}{\sigma_{X}\sigma_{Y}}=\;\frac{E\left[\left(X-\mu_{X}\right)\left(Y-\mu_{Y}\right)\right]}{\sigma_{X}\sigma_{Y}}$$

The above equation defines the overall correlation coefficient, and the Greek lowercase letters are commonly used as representative symbols. To estimate the covariance and standard deviation of the sample, the Pearson correlation coefficient (r). *P* values of all statistical processes were bilateral, with *P* values< 0.05 considered significant.

## 3 Results

### 3.1 Workflow

**Figure 1:**
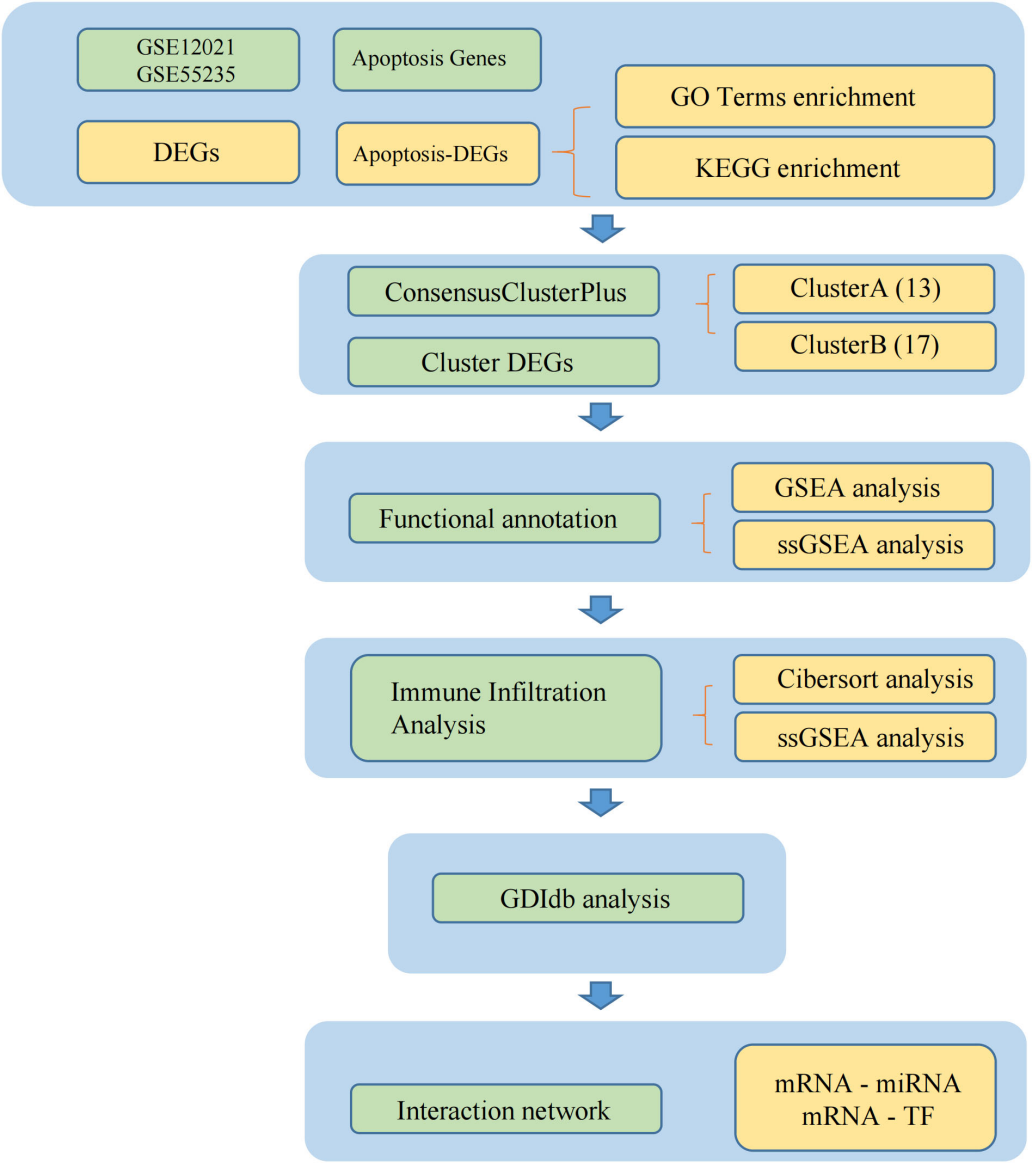
The flow chart.

The workflow is depicted in **Figure 1**.

### 3.2 Data set combination, OA-related DEGs, and their GO functional enrichment

**Figure 2:**
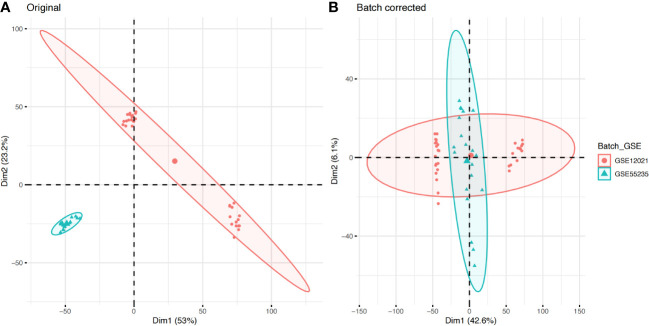
Gene expression distribution and batch effect correction of all samples. **(A)** The difference between the two data sets before removing the batch effect. **(B)** The difference between the two data sets after removing the batch effect.

**Figure 3:**
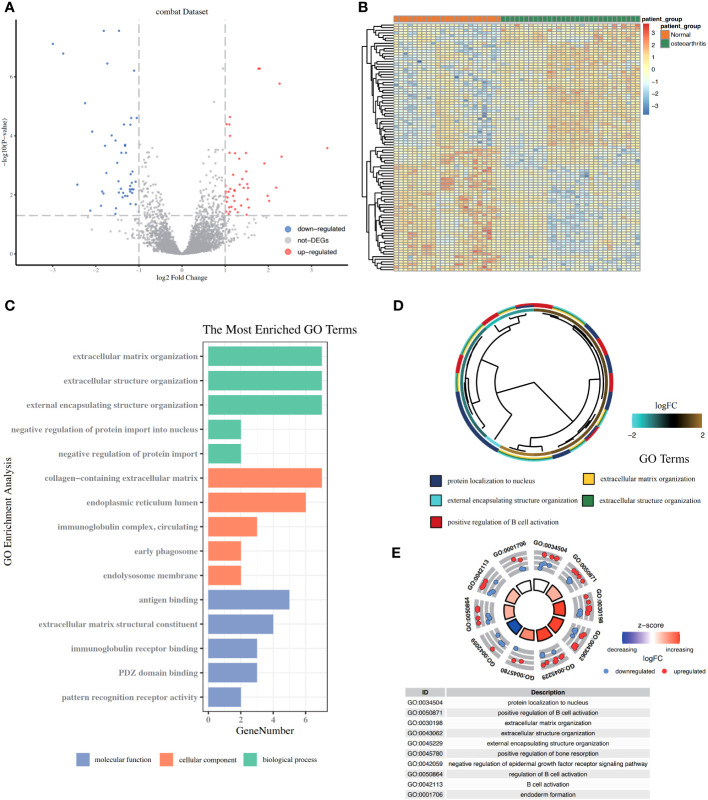
The ARDEGs. **(A)** The volcano map of DEGs related to osteoarthritis (OA). The x-axis represents log2FoldChange, and the y-axis represents -log10 (P-value). The red node represents the upregulated differentially expressed genes (DEGs), blue node represents the downregulated DEGs, and the gray node represents the non-significant genes. **(B)** Heat map of the expression level of DEGs related to OA; green represents disease samples, brown represents normal control samples, red represents high expression, and blue represents low expression. **(C–E)** Functional enrichment analysis of DEGs. **(C)** The abscissa represents the enriched gene book, the ordinate represents the biological process, and the color indicates the significance of the enrichment results. **(D)** Ring diagram of GO enrichment results. The innermost ring in the figure is the gene cluster tree. The middle ring is the logFC value corresponding to these genes. The closer the red logFC value, the higher the upregulation level. The outer ring is the GO terms enriched by these genes. Each GO term uses a color to set itself apart. **(E)** The result of the enrichment analysis of the KEGG pathway shows that the node color represents the gene expression level, and the quadrilateral color represents the KEGG pathway Z-core. ARDEGs, apoptosis-related differentially expressed genes; DEGs, differentially expressed genes.

In this study, the two data sets GSE12021 and GSE55235 were combined, and the batch effect between the data sets was removed (**Figure 2**). Ninety-five DEGs were then obtained from the OA (n = 30) and control samples (n = 23), including 47 upregulated and 48 downregulated genes (**Figures 3A, B**). GO functional annotation was performed on the DEGs to determine their biological functions (**Figures 3C–E**). The results showed that these DEGs were mainly enriched in BPs, such as ECM organization, extracellular structure organization, and CCs, such as collagen-containing extracellular matrix, immunoglobulin complex/circulating, as well as MFs such as immunoglobulin receptor binding, antigen binding (**Figures 3C–E**).

### 3.3 Risk prediction model construction

**Figure 4:**
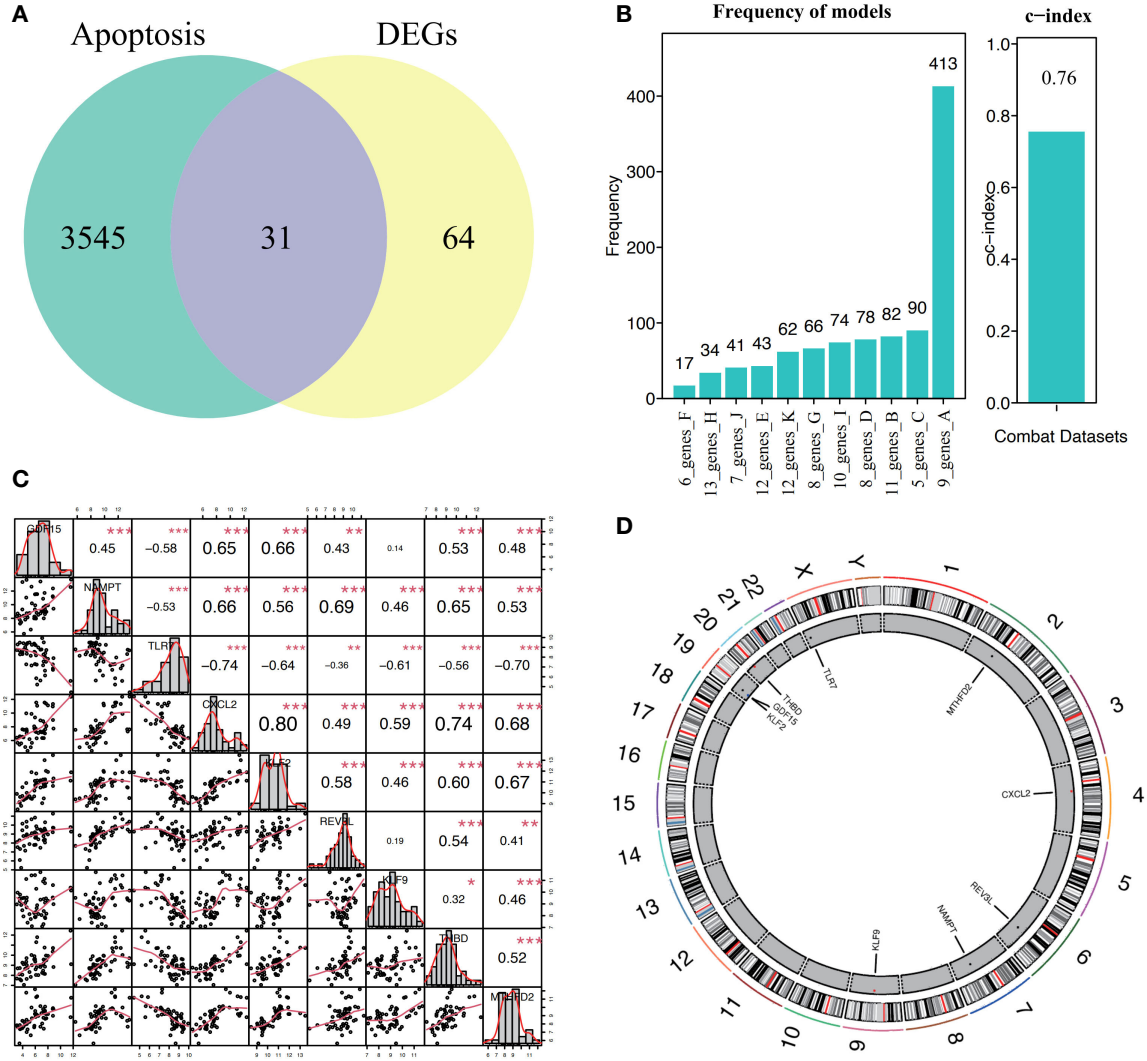
Construction and correlation analysis of the diagnosis model of osteoarthritis. **(A, B)** Through lasso algorithm, characteristic genes are selected from apoptosis-related genes. The horizontal axis is the combination of genes selected through 1000 Lasso analyses, and the vertical axis is the number of occurrences. **(C)** For the correlation analysis of nine characteristic genes in all samples, *P< 0.05, **P< 0.01, ***P< 0.001, and the number represents the correlation level. **(D)** Location map of nine characteristic genes on the chromosome.

A total of 31 hub genes were selected from the apoptotic genes and DEGs (**Figure 4A**), and hub genes with FDR< 0.05 were selected as candidate genes. The hub genes were put into logistic Lasso analysis. The analysis results revealed nine characteristic genes, namely growth differentiation factor 15 (*GDF15), NAMPT, TLR7, CXCL2, KLF2, REV3L, KLF9, THBD*, and *MTHFD2*, which occurred 413 times in 1,000 cycles (**Figure 4B**; **Table 1**). The gene correlations of the nine characteristic genes were calculated. The results showed a strong correlation between *CXCL2* and *KLF2* (r = 0.8, *P*< 0.05, r for the correlation coefficient), also *CXCL2* and *THBD* (r = 0.74, *P*< 0.05) (**Figure 4C**). The location of 18 genes on the human chromosome through the R package RCircos is shown (**Figure 4D**), and the results showed that these genes were frequently found on chr2, chr4, chr6, chr7, chr9, chr19, chr20, and chrX.

**Table 1:** The gene lists.

Gene	
CXCL2	
GDF15	
KLF2	
KLF9	
MTHFD2	
NAMPT	
REV3L	
THBD	
TLR7	

**Figure 5:**
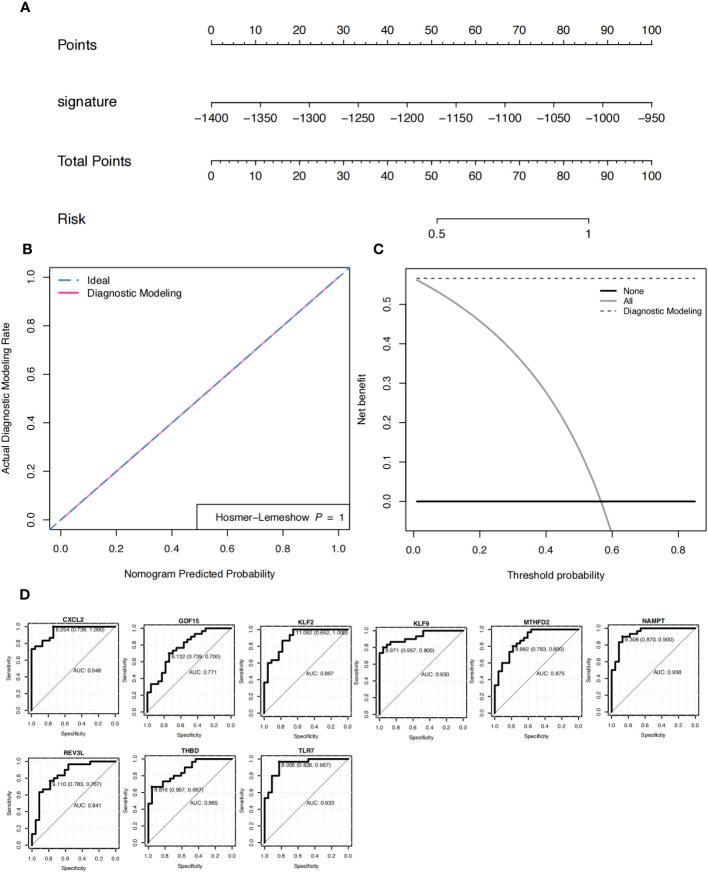
Nomogram. **(A)** Nomograms predicting the risk score for the diagnosis of patients with osteoarthritis (OA). **(B)** The model evaluation curve; gray represents random diagnosis, while green and pink represent model diagnosis. **(C)** DCA curve. The x-axis represents the risk threshold, y-axis represents the net profit rate, black solid line represents 0 net profit rate, all (gray solid line) represents that all samples are subject to intervention, and the affected site modeling (black dotted line) represents the model curve. **(D)** ROC curve of nine characteristic genes in the diagnosis of OA.

Based on the patient’s predicted risk score for OA, the results showed that the score had a profound effect (**Figure 5A**). The calibration curve indicated that the nomogram model was accurate (**Figure 5B**). Decision curve analysis (DCA) was used to evaluate the potential clinical impact of the nomogram model and its association with OA. The dashed line in the DCA curve remained above the grey and black lines from 0 to 1, indicating that the decision based on the nomogram model may be beneficial for patients with OA (**Figure 5C**). Simultaneously, the ROC curves of the nine characteristic genes were analyzed to predict OA independently, and the results showed that the nine characteristic genes had an efficient predictive ability (**Figure 5D**).

### 3.4 Apoptosis mode identification

**Figure 6:**
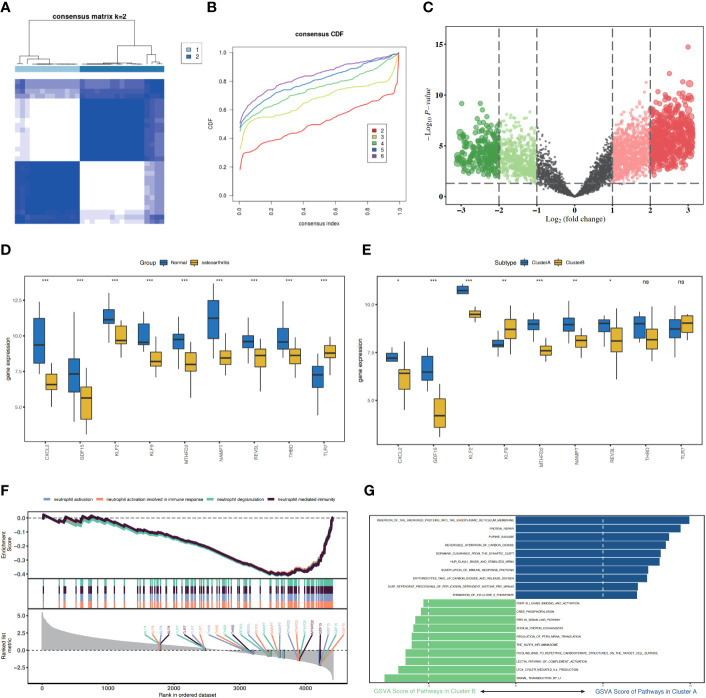
Consistency cluster score of patients with osteoarthritis (OA) and functional analysis between the two apoptosis modes. **(A)** Consistency clustering result diagram. **(B)** CDF, this figure shows the cumulative distribution function when K takes different values. It is used to judge the value of K, and CDF reaches the approximate maximum value. During this time, the cluster analysis results are the most reliable, usually taking the value of K with a small decline slope of CDF. **(C)** Cluster A and Cluster B differentially expressed genes (DEGs) have a volcanic map with log2FoldChange as the abscissa and -log10 (P-value) as the ordinate. The red node represents the upregulated DEGs, green node represents the downregulated DEGs, and gray node represents the cause of non-significant DEGs. **(D)** Histograms of the expression levels of nine characteristic genes in the disease group and the control group, with blue indicating the control group and yellow indicating the disease group. **(E)** Histogram of the expression level of nine characteristic genes in ClusterA and ClusterB of the OA subgroups, blue represents ClusterA, and yellow represents ClusterB. **(F)** GSEA analysis results between ClusterA and ClusterB. **(G)** SSGSEA analysis of Cluster A and Cluster B, KEGG biological function enrichment analysis. CDF, cumulative distribution function; SSGSEA, single sample gene set enrichment analysis. * represents P < 0.1, ** represents P < 0.01, *** represents P < 0.001, ns represents non-significant.

Using the “ConsensusClusterPlus” package in the R software, based on nine characteristic genes, two cell apoptosis subtypes (ClusterA and ClusterB) were identified using the consistency clustering method (**Figures 6A, B**). The volcanic map of DEGs of Cluster A and Cluster B was shown in the **Figure 6C**. Except for *TLR7*, the nine characteristic genes were expressed at high levels in the normal samples. ClusterA contained 13 samples and ClusterB contained 17 samples. The expression levels of the characteristic genes *CXCL2, GDF15, KLF2, MTHFD2, NAMPT, and REV3L* were significantly higher in ClusterA than those in ClusterB, whereas the expression level of *KLF9* was higher in ClusterB than that in ClusterA (**Figures 6D, E**).

### 3.5 Analysis of functional differences between two different cell apoptosis modes

To analyze the differences between the two different cell apoptosis modes, DEGs were obtained in the two cell apoptosis modes, ClusterA and ClusterB (**Figure 6F**). We then analyzed the effects of DEGs between the two cell apoptosis modes on the biological functions of patients. GSEA revealed that *neutrophil activation* and *neutrophil activation involved in immune response* were highly enriched in ClusterB (**Figure 6F**). Using ssGSEA, we explored the highly enriched biological signaling pathways in the two cell apoptosis subgroups. The results showed that the protein repair and purine salvage signaling pathways were highly enriched in the ClusterA model (**Figure 6G**).

### 3.6 Differences in immune characteristics between the two cell apoptosis modes

**Figure 7:**
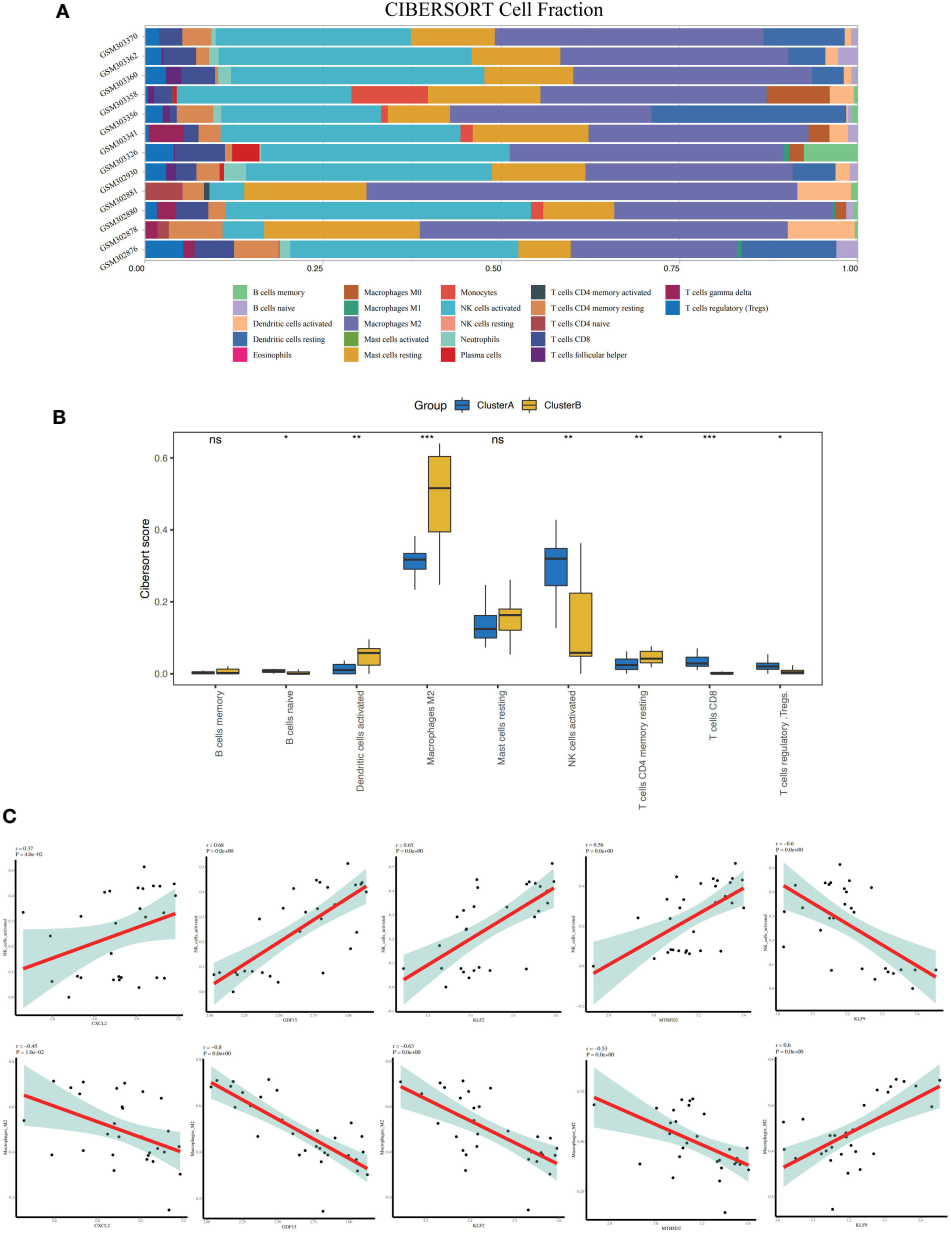
Immune characteristics between the two apoptosis modes - CIBERSORT. **(A)** Accumulation diagram of immune cell content between Cluster A and Cluster B shows different immune cells in different colors, and the x-axis represents the cell fraction and the y-axis represents the sample name. **(B)** Histogram of the content of immune cells in patients of Cluster A and Cluster B; blue represents the Cluster A sample, and yellow represents the Cluster B sample. **(C)** Correlation between nine characteristic genes and immune cells. The x-axis represents the amount of gene expression, and the y-axis represents score of immune cell infiltration. r > 0 represents a positive correlation and r< 0 represents a negative correlation. * represents P < 0.1, ** represents P < 0.01, *** represents P < 0.001, ns represents non-significant.

The results of the CIBERSORT analysis showed that the level of activated natural killer (NK) cells in ClusterB was significantly higher than that in ClusterA. The M2 macrophage content in patients in ClusterA was significantly higher than that in ClusterB (**Figures 7A, B**).

### 3.7 Analysis of correlation between key genes and immune cell infiltration

The results showed that the expression of *CXCL2, GDF15, KLF2*, and *MTHFD2* genes was positively correlated with activated NK cells (r = 0.37, *P* = 0.04; r = 0.68, *P*< 0.01; r = 0.65, *P*< 0.01; R = 0.56, *P*< 0.01, respectively), whereas the gene expression of *KLF9* was negatively correlated with activated NK cells (r = -0.6, *P*< 0.01) (**Figure 7C**). The expression of *CXCL2, GDF15, KLF2*, and *MTHFD2* genes was negatively correlated with M2 macrophages (r = -0.45, *P* = 0.01; r = -0.8, *P*< 0.01; r = -0.63, *P*< 0.01; r = -0.53, *P*< 0.01), whereas the gene expression of *KLF9* was positively correlated with M2 macrophages (r = 0.6, *P<* 0.01) (**Figure 7C**).

**Figure 8:**
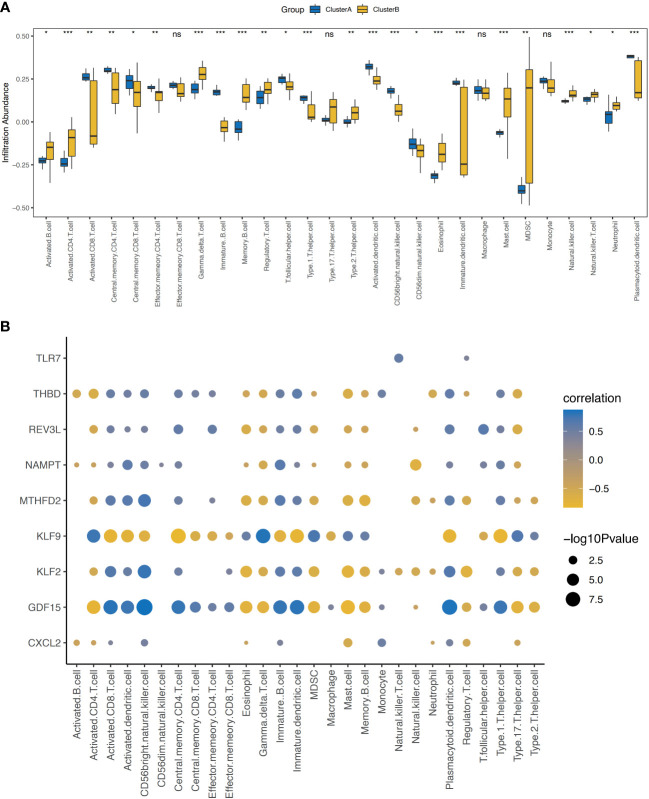
Immune characteristics between the two cell apoptosis modes - ssGSEA. **(A)** Histogram of the content of immune cells in patients of Cluster A and Cluster B; yellow represents the Cluster B sample and blue represents the Cluster A sample. **(B)** Correlation analysis between the nine characteristic genes and the content of immune cells shows that the x-axis represents the immune cells, y-axis represents the characteristic genes, node color represents the correlation size, and node size represents the significance level. ssGEA, single-sample gene set enrichment analysis. * represents P < 0.1, ** represents P < 0.01, *** represents P < 0.001, ns represents non-significant.

The ssGSEA results showed that the contents of several immune cells in ClusterA were higher than those in ClusterB, such as the activated CD8 T cell, the central memory CD4 T cell, the central memory CD8 T cell, and the Type 1 T helper cell (P< 0.05). Nevertheless, the contents of some immune cells in ClusterA were lower than those in ClusterB, such as regulatory T cell, the natural killer cell, the gamma delta T cell, Type 2 T helper cell, the neutrophil, and nature killer T cell (P< 0.05, **Figure 8A**). Correlation analysis showed that the nine characteristic genes were closely related to immune cell content (**Figure 8B**).

**Figure 9:**
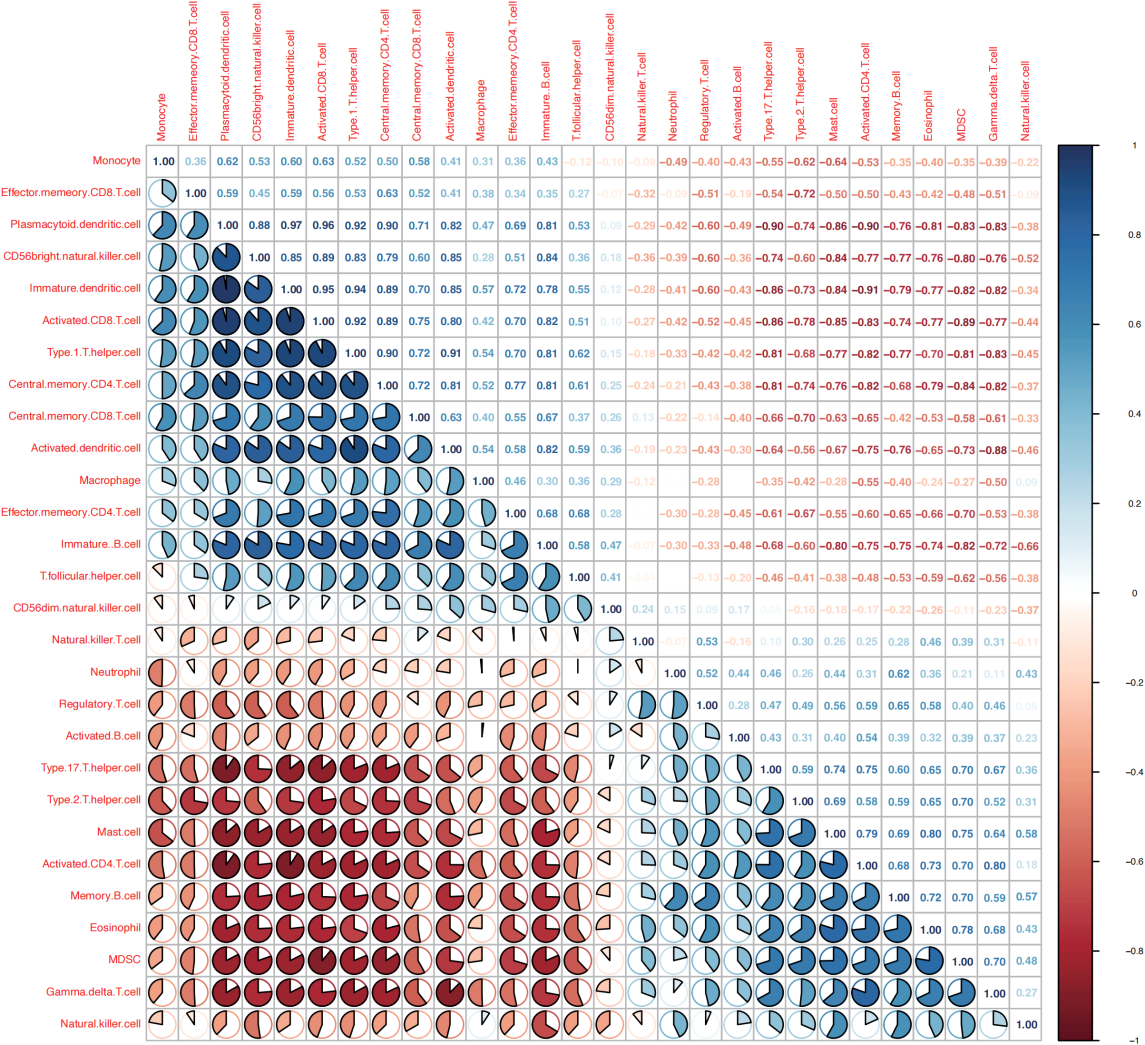
Correlation between immune cells in patients with osteoarthritis (OA). The correlation of immune cells in patients with OA is negative in red and positive in blue.

As shown in **Figure 9**, the correlations between the immune cells in patients with OA was further calculated, the results showed that NK cells showed significantly positively correlated with gamma delta T cells, MDSC, and eosinophils (P< 0.05), whereas negatively correlated with immature B cells, macrophages, and activated dendritic cells (P< 0.05).3.8 Drug–gene interaction analysis.

**Figure 10:**
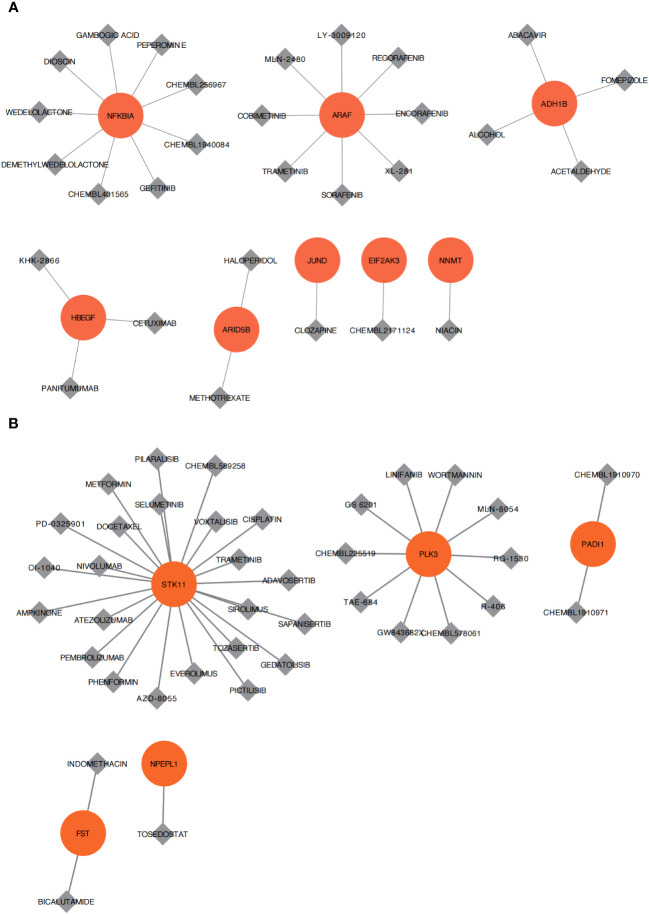
Drug sensitivity analysis. **(A)** Cell apoptosis of osteoarthritis is related to small molecular compounds or drug sensitivity analysis of Cluster A. **(B)** Cell apoptosis of osteoarthritis is related to small molecular compounds or drug sensitivity analysis of Cluster B. Red represents the core differential genes of each subtype, gray represents small molecular compounds or drugs, and the connection represents the connection between drugs and genes.

The DGIdb database was used to retrieve small-molecule compounds and drugs that regulated key genes. As shown in the drug–gene interaction network, 29 drugs or molecular compounds, including mepyrazole, conafinil, and abacavir, were associated with the key genes *NFKBIA, ARAF*, and *ADH1B* of OA ClusterA (**Figure 10A**). A total of 38 drugs or molecular compounds, including PICTILISIB, Linifanib, and CHEMBL225519, were related to the key genes *STK11, PLK3*, and *PADI1* of OA ClusterB (**Figure 10B**). The relevance of these drugs or compounds to key genes could imply that they may have varying degrees of effect in regulating these genes.

### 3.9 PPI network

**Figure 11:**
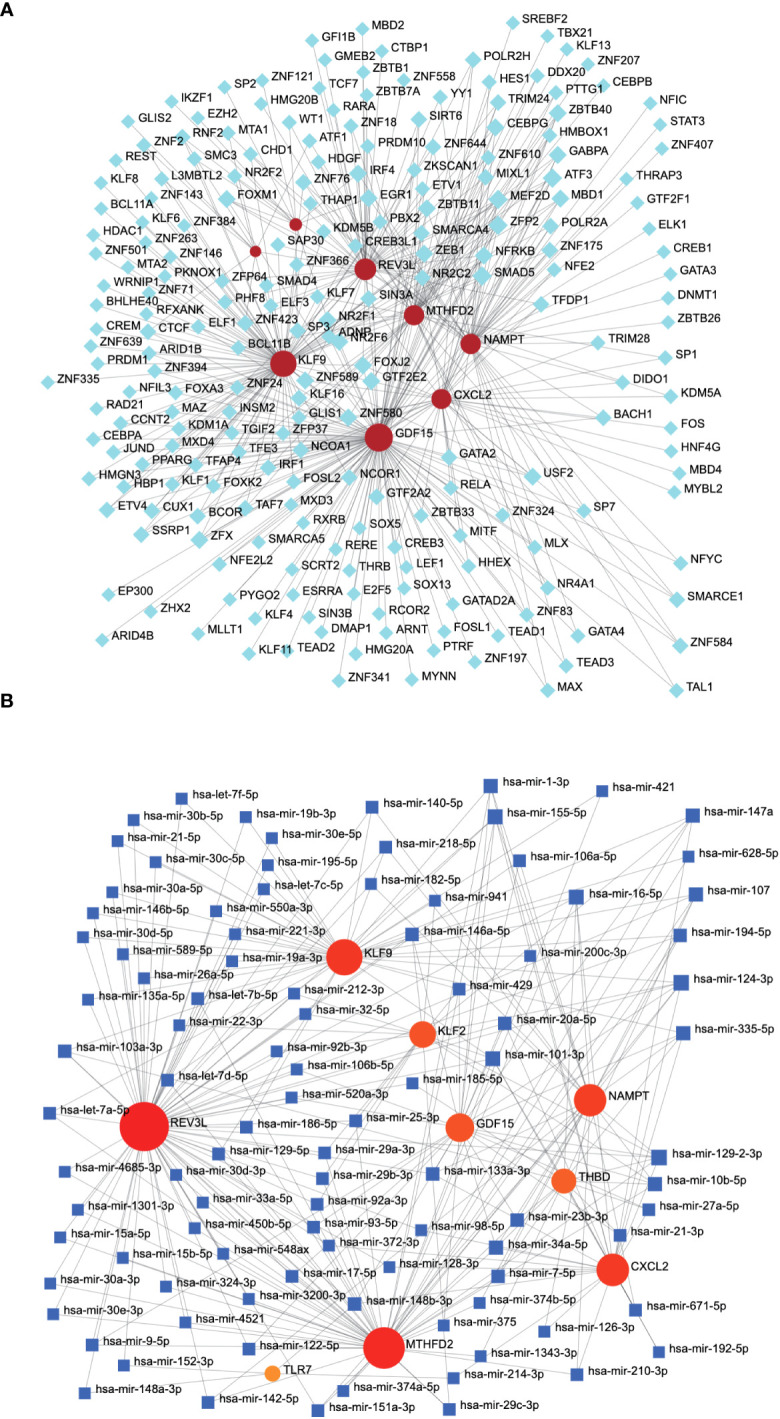
mRNA–miRNA and mRNA–TF network associated with characteristic genes. **(A)** The mRNA–TF network related to characteristic genes, with gray nodes representing TF and red nodes representing characteristic genes. **(B)** The mRNA-miRNA network associated with the characteristic gene. The gray node represents the miRNA and the blue node represents the characteristic gene. TF, transcription factor.

We constructed mRNA–miRNA and mRNA–TF networks of the characteristic genes. The mRNA-TF network included 1,324 interactions, 8 mRNA, and 368 TFs. *GDF15* interacted with 193 TFs, while *KLF9* interacted with 232 TFs (**Figure 11A**). The mRNA–miRNA network of the nine characteristic genes was constructed, including 4,213 interaction relationships, 9 mRNAs, and 1,236 miRNAs, with *CXCL2* interacting with 48 miRNAs and *REV3L* interacting with 82 miRNAs (**Figure 11B**).

## 4 Discussion

OA is the most common chronic degenerative joint disease encountered in orthopedic clinics, among which the most commonly affected joints are the hip and knee joints. The incidence rate of OA increases annually with increasing age, especially in middle-aged and older populations (28). In addition to relieving symptoms, there are currently no reports confirming that existing treatment measures can prevent or reverse OA progression. Therefore, the pathogenesis of atherosclerosis has become a popular research topic. OA is the result of multiple factors, such as cartilage nutrition, metabolic abnormalities, stress imbalance, abnormal degradation of the cartilage matrix by enzymes, cumulative minor trauma, obesity, and increased joint weight bearing (29). The occurrence of OA is a continuous process, and its basic pathological mechanism involves cartilage degeneration and erosion. As the disease continues to develop, cystic changes in the subchondral bone occur gradually, and bone spurs begin to appear at the edge of the joint. In this continuous pathological change, cartilage degeneration is a key link and the initiating factor in the occurrence and development of OA (30). In addition to relieving the symptoms of OA, there are currently no reports confirming whether the existing treatment measures can prevent or reverse its progression. Therefore, studying OA pathogenesis and progression is essential for understanding the disease and proposing effective treatment targets.

Some researchers believe that OA development is closely related to chondrocyte apoptosis. Hwang et al. (31) suggested that significant apoptosis occurs in advanced OA, resulting in a decrease in chondrocytes, often accompanied by lacunar emptying, highlighting that chondrocyte apoptosis is a characteristic of OA progression. However, the role of apoptosis in the progressive degeneration of cartilage remains unclear. Identifying key genes and pathways related to cell apoptosis can help people understand the process of OA occurrence and development and carry out targeted molecular research to design targeted therapeutic drugs.

Therefore, to analyze the correlation between apoptosis and OA development, we selected apoptosis genes from the DEGs between OA and normal samples and performed LASSO regression analysis to identify nine characteristic genes: *GDF15, NAMPT, TLR7, CXCL2, KLF2, REV3L, KLF9, THBD*, and *MTHFD2*. We also constructed a risk-prediction nomogram. Testing the prediction accuracy of the nomogram showed that nine apoptotic genes accurately predicted OA occurrence. This indicated that apoptosis and apoptotic genes play key roles in OA occurrence and development.

*GDF15*, a member of the tumor growth factor superfamily, has recently been identified as a possible biomarker of aging and is associated with various clinical conditions, including coronary artery disease, diabetes, and various cancer types. Wen et al. (32) studied the role of the *GDF15*/MAPK14 axis in the aging of chondrocytes in OA and found that *GDF15* is a driving factor for chondrocyte aging and apoptosis and could promote the progression of OA by inducing angiogenesis. *TLR7* is involved in immune responses in many inflammatory diseases. Liu et al. (33) believe that silencing *TLR7* could block the p21-mediated JAK2/STAT3 pathway and prevent lipopolysaccharide-induced apoptosis and chondrocyte injury. The results of the present study are consistent with those of these studies.

We conducted a differential analysis using OA samples and control samples to obtain 95 DEGs, which were mainly enriched in ECM organization, extracellular structure organization, collagen-containing ECM, immunoglobulin complex, circulation, immunoglobulin circulating, immunoglobulin-receiver-binding, and antigen binding. Some researchers have suggested that with the progression of the disease, the immune barrier of the ECM disappears and specific surface antigens of chondrocytes cause autoimmune reactions, opening a molecular damage mode for ECM products. The innate immune and inflammatory cycle mechanisms in OA lead to sustained joint damage (34). The results of our genetic enrichment analysis are consistent with these findings.

Previous studies have found rare mutations in certain genes that can lead to hereditary OA, even results in severe OA (35–37). At least 100 risk loci associated with OA have also been identified by genome wide-association studies (38, 39). In this study, we plotted the genetic locus on the chromosome of characteristic genes. These results provide clues for further studies on the genetic underpinnings and biological mechanisms of OA pathogenesis. First, neighboring genes in the same chromosomal regions as the potentially OA-related genes may participate in similar pathways and functions. Second, further studies will focus on exploring the relationships between single nucleotide polymorphisms in these genes and OA risk. Furthermore, the overlap between gene locations and regulatory elements suggests these genes could be co-regulated in certain biological processes important to OA. In our study, differential expression gene analysis revealed interesting patterns, such as KLF2 and GDF15 genes located on the same chromosome. The observation of differential genes clustered on specific chromosomes has prompted us to delve deeper into the potential implications of this phenomenon. The co-localization of differentially expressed genes on the same chromosome may reflect a coordinated regulatory mechanism that regulate their expression levels. This co-regulation may be attributed to various factors, such as shared transcriptional regulatory elements, epigenetic modifications, and even physical chromatin interactions. By enhancing our understanding of OA genetics and biology, these findings could ultimately promote drug development.

To subdivide the different pathways and characteristics of cell apoptosis, we attempted to use nine genes to perform a consensus cluster analysis on 30 OA samples and finally obtained two clusters, namely Cluster A and Cluster B, including 13 samples for ClusterA and 17 samples for Cluster B. The expression of nine characteristic genes in both clusters was significantly increased or decreased. We compared the biologically related functions and pathways of the two clusters and found that cluster A was highly enriched in protein repair and purine salvage signaling pathways, whereas Cluster B was highly enriched in neutrophil activation, neutrophil activation involved in immune response, and other neutrophil-related pathways in the cluster B subgroup. These results suggest that cluster A was highly enriched in cell repair and rescue, and cluster B was involved in inflammation-related immune responses. Immune cell infiltration analysis showed that the expression levels of T cells and M2 macrophages was higher in cluster A, whereas the expression levels of activated NK cells was significantly higher in cluster B. This reflects the different immune microenvironments of the two types of samples. We speculated that this difference in immune cell infiltration may be related to the different OA progression in the two types of samples. Chondrocyte aging and apoptosis play a promoting role during OA occurrence and development, (Cluster B). At this stage, the patient’s immune response is dominated by adaptive immunity, manifested by the disappearance of the ECM immune barrier and the release of specific surface antigens from chondrocytes, which stimulate the corresponding T cells in the lymphocyte pool of the immune system, triggering the process of cell-mediated immunity. In this process, T cells release lymphatic factors to clear antigens, causing damage to the cartilage cells and initiating BPs, such as protein repair and purine rescue. However, the high level of NK cells is related to the clearance of inflammatory factors, and the high concentration of neutrophil-related pathways in cluster A suggests that the patients have progressed to the stage of inflammatory circulation, and its immune response is dominated by innate immunity, with an increase in the level of NK cells as a significant feature.

We also observed that the expression levels of the *CXCL2, GDF15, KLF2*, and *MTHFD2* genes were positively correlated with the number of activated NK cells and negatively correlated with the number of M2 macrophages; however, the reverse was true for *KLF9*. Therefore, we speculate that these five genes are involved in regulating the immune response. Evidence for this can be found in various literature. *CXCL2* is a member of the chemokine superfamily that encodes secreted proteins involved in immune regulation and inflammatory processes. This chemokine is a member of the CXC subfamily, expressed at inflammatory sites, and may inhibit the proliferation of hematopoietic progenitor cells (40). *KLF2* regulates innate immune responses during skeletal muscle injury and regeneration (41). Wang et al. (42) confirmed that *GDF15* induces immunosuppression through regulatory T cells.

In colon adenocarcinoma, patients with high GDF15 had favorable overall survival, but this survival advantage was reversed when they also had decreased NK cells (43). Another study suggested that decidual NK cells contribute to embryo growth by GDF15 secretion (44). Besides, GDF15 overexpression was associated with increased *CD8* T cell numbers and proportion of activated CD8*+*CD11c+ T cells. Furthermore, depletion of *CD8* T cells in tumor-bearing mice eliminated the protective effect of *GDF15* against tumor growth (45). In accordance with these observations, our results suggested that both GDF15 and NK cells and activated CD8 T cells were significantly increased in Cluster A, and they were positively correlated. Further investigations with some wet lab evidences are needed to validate our data and find more meaningful results.

According to our results, there were many changes and correlations among immune cell subpopulations, which indicated that various immune cells contribute to pathogenesis of OA through complex mechanisms. Macrophages, B cells, T cells, and NK cells produced proinflammatory cytokines like interleukin-1β (IL-1β) (46). which induces extracellular matrix degradation (47) and chondrocyte apoptosis by promoting chondrocyte hypertrophy and dedifferentiation (31). It has been reported that Th17 cells drive OA progression by stimulating osteoclast progenitor recruitment through increased chemokine production from bone marrow mesenchymal stromal cells (48). In the patients with OA, the number of CD4+ T cell is elevated in the subsynovial layer compared to healthy group (49). CD4+ T cell secret interferon-gamma (IFN-γ) which can promote mesenchymal stem cell differentiation, while transforming growth factor-β (TGF-β) from these cells is negatively related to osteoblast differentiation (50). Distinct CD4+ T helper cell subsets also influence joint inflammation and remodel. Th1 cells promote immune response via producing IFN-γ- and IL-2-related cytokines (51). IL-4 released by Th2 cells exerts protective effects on cartilage (49). Additionally, memory T cells impair immune function (52). Targeting these various immune cell types and their signaling molecules may be helpful to clarify the pathogenesis of OA and explore new therapeutic strategies for managing OA.

Treg cells have been found to participate in changes in subchondral bone remodeling, an important process in the pathogenesis of OA. Treg cells secret various of cytokines and further promote osteoclast maturation (53). However, Treg cells also secrete IL-17F, IL-17A and BMP-2 that strongly promote osteoblastic differentiation (54). Additionally, Treg cells can inhibit bone resorption by promoting osteoclast apoptosis (55). While Treg cells have complex effects on bone remodeling through multiple mechanisms, the exactly biological mechanisms require further exploration.

T helper (Th) cells interact with other immune cells, such as B lymphocytes, through cytokine signaling and participate in immune regulation (56). B lymphocytes can activate T cells by presenting antigens like granulocyte colony-stimulating factor (G-CSF) (57), which promotes osteoclast progenitor proliferation (58). Inflammatory mediators secreted by neutrophils and osteoclasts differentially impact mesenchymal stem cells (MSCs) (59). MSCs can inhibit NK cell cytotoxicity and proliferation, prevent autoreactive antibody production, suppress Th1 cell activation, and stimulate Treg cell generation (60). Additionally, macrophages communicate with osteocytes through paracrine signaling and direct cell-cell contact (61). Taken together, interactions among various immune cell types including T cells, B cells, neutrophils, osteoclasts, MSCs, and macrophages influence bone remodeling and the progression of OA.

In drug sensitivity analysis, we compared the differential genes of two modes of cell apoptosis (Cluster A and Cluster B) and attempted to find targeted therapeutic drugs. We found 29 molecular compounds targeting key genes of Cluster A and 38 molecular compounds targeting Cluster B. Although these drugs were rarely used in the clinical treatment of osteoarthritis, they may become potential treatment agents, which remain to be discovered in the future studies.

This study has some limitations. First, although there are significant differences in the immune characteristics and functional enrichment between Cluster A and Cluster B, we have not yet found an exact grade of OA to correspond to the clusters; second, our data were from GEO. The total sample size for OA was only 30 cases, which is relatively small, and a data bias may have occurred during the research process. Third, we did not directly identify specific drugs or molecules that regulate the nine characteristic genes that affect OA progression, which should be further explored in future studies.

Apoptotic genes play a key role in the development and occurrence of OA. The ARGs *GDF15, NAMPT, TLR7, CXCL2, KLF2, REV3L, KLF9, THBD*, and *MTHFD2* could independently and accurately predict OA occurrence. Patients with OA can be divided into two clusters with significant differences in immune characteristics, functional enrichment, and immune infiltration, which may be related to different degrees of OA progression. However, more clinical evidence is needed to confirm this. *CXCL2, GDF15, KLF2, MTHFD2*, and *KLF9* are strongly correlated with immune infiltration in patients with OA and could become novel therapeutic targets that affect OA progression.

## Data availability statement

The original contributions presented in the study are publicly available. This data can be found here: https://www.jianguoyun.com/p/DbKXe40Q1ujHCxig64IFIAA.

## Author contributions

EY and JY conceived and designed the research; EY, MZ, GX and XL performed the research, analyzed the data, and prepared figures; JY wrote the paper. Al authors listed have made a substantial, direct, and intellectual contribution to the work and approved it for publication.
